# Antioxidant, Enzyme Inhibitory, and Molecular Docking Approaches to the Antidiabetic Potentials of Bioactive Compounds from *Persicaria hydropiper* L.

**DOI:** 10.1155/2022/6705810

**Published:** 2022-04-14

**Authors:** Muhammad Ayaz, Abdul Sadiq, Osama F. Mosa, Tariq Abdalla Zafar, Alashary Adam Eisa Hamdoon, Modawy Elnour Modawy Elkhalifa, Mohammed Ahmed Elawad, Alshebli Ahmed, Farhat Ullah, Mehreen Ghufran, Atul Kabra

**Affiliations:** ^1^Department of Pharmacy, Faculty of Biological Sciences, University of Malakand, Chakdara 18000, Dir (L), KP, Pakistan; ^2^Public Health Department Health Sciences College at Lieth, Umm Al Qura University, Makkah, Saudi Arabia; ^3^Biochemistry Department, Bukhara State Medical Institute Named after Abu Ali Ibn Sino, Bukhara, Uzbekistan; ^4^Faculty of Public and Environmental Health, University of Khartoum, Khartoum, Sudan; ^5^Department of Pathology, MTI Bacha Khan Medical College, Mardan, Pakistan; ^6^University Institute of Pharma Sciences, Chandigarh University, Gharuan, Mohali-140413, Punjab, India

## Abstract

**Introduction:**

Natural products are among the most useful sources for the discovery of new drugs against various diseases. Keeping in view the ethnobotanical relevance ethnopharmacological significance of Polygonaceae family in diabetes, the current study was designed to isolate pure compounds from *Persicaria hydropiper* L. leaves and evaluate their *in vitro* and *in silico* antidiabetic potentials.

**Methods:**

Six compounds were isolated from the chloroform-ethyl acetate fractions using gravity column chromatography and were subjected to structure elucidation process. Structures were confirmed using ^1^H-NMR, ^13^C-NMR, and mass spectrometry techniques. Isolated phytochemicals were subjected to *in vitro* antidiabetic studies, including *α*-glucosidase, *α*-amylase inhibition, and DPPH, and ABTS antioxidant studies. Furthermore, the *in silico* binding mode of these compounds in the target enzymes was elucidated via MOE-Dock software.

**Results:**

The isolated compounds revealed concentration-dependent inhibitions against *α*-glucosidase enzyme. Ph-1 and Ph-2 were most potent with 81.84 and 78.79% enzyme inhibitions at 1000 *µ*g·mL^−1^, respectively. Ph-1 and Ph-2 exhibited IC_50_s of 85 and 170 *µ*g·mL^−1^ correspondingly. Likewise, test compounds showed considerable *α*-amylase inhibitions with Ph-1 and Ph-2 being the most potent. Tested compounds exhibited considerable antioxidant potentials in both DPPH and ABTS assays. Molecular simulation studies also revealed top-ranked confirmations for the majority of the compounds in the target enzymes. Highest observed potent compound was Ph-1 with docking score of −12.4286 and formed eight hydrogen bonds and three H-pi linkages with the Asp 68, Phe 157, Phe 177, Asn 241, Glu 276, His 279, Phe 300, Glu 304, Ser 308, Pro 309, Phe 310, Asp 349, and Arg 439 residues of *α*-glucosidase binding packets. Asp 68, Glu 276, Asp 349, and Arg 439 formed polar bonds with the 3-ethyl-2-methylpentane moiety of the ligand.

**Conclusions:**

The isolated compounds exhibited considerable antioxidant and inhibitory potentials against vital enzymes implicated in T2DM. The docking scores of the compounds revealed that they exhibit affinity for binding with target ligands. The enzyme inhibition and antioxidant potential of the compounds might contribute to the hypoglycemic effects of the plant and need further studies.

## 1. Introduction

Diabetes mellitus (DM) is a metabolic syndrome characterized by hyperglycemia and other complications including nephropathy, neuropathy, retinopathy, and micro- and macro-vascular complications [1]. It is among the highly prevalent diseases and is estimated to affect about 300 million people globally by 2025 [2]. The preliminary stages of non-insulin-dependent diabetes is allied with postprandial hyperglycemia owing to impaired insulin secretion after meal. Among the therapeutic approaches for type 2 diabetes (T2DM) is to retard glucose absorption from gastrointestinal tract. *α*-Glucosidase (*α*-D-glucoside glucohydrolase) enzyme is involved in the metabolism of dietary carbohydrates to form simple monosaccharides via the catalytic liberation of *α*-glucose from the nonreducing end of the substrate. *α*-Amylase enzyme is also implicated in the metabolic breakdown of long chain carbohydrates and thus help in their gastrointestinal absorption [3, 4]. Inhibitors of these enzymes diminish the hydrolytic cleavage of oligosaccharides and thus delay their absorption from intestinal tract [5]. This is scientifically proved to be one of the best approaches to reduce the postprandial rise in blood glucose in order to avoid the inception of late diabetic complications [6, 7]. Consequently, these enzymes are important drug targets in the prevention of T2DM, which reduce the after-meal glucose level in the blood.

Preclinical and clinical antidiabetic studies on medicinal plants revealed their greater potential for drug discovery against diabetes. *Galega officinalis* L. from Fabaceae family was the first-ever medicinal plant with a clear antidiabetic profile and has a long history of use as a complementary antidiabetic therapy. Later, an important antidiabetic compound galegine was isolated from this plant that has almost similar structure with its synthetic counterpart metformin [8, 9]. Plants' secondary metabolites including flavonoids and anthocyanins have been extensively reported for antidiabetic potentials. For instance, anthocyanins isolated from *Pharbitis nil* L. and *Ipomoea batatas* L. significantly reduce postprandial hyperglycemia via inhibition of *α*-glucosidase enzyme [7]. Likewise, scientific validation of a polyphenols-rich plant *Pinus pinaster* leads to the discovery of an important *α*-glucosidase and competitive *α*-amylase inhibitor pycnogenol [10, 11]. Additionally, acarbose, miglitol, and voglibose are important *α*-glucosidase and *α*-amylase inhibitors isolated from microbial origin [12]. Numerous studies on crude extracts and isolated pure compounds signify the role of natural resources in the discovery and development of safer and more effective antidiabetic remedies.

*Persicaria hydropiper* L. belong to family Polygonaceae (smartweed), which consist of about fifty genera and twelve hundred species [13]. Polygonaceae family has a long history of ethnomedicinal antidiabetic potentials. *Antigonon leptopus* Hook. & Arn., a member of Polygonaceae, is used in traditional medicine to treat hyperglycemia and is scientifically validated in animal models [14, 15]. Preliminary studies on *P. hydropiper* L. ethanolic extract revealed antidiabetic potentials in oral glucose tolerance test [16]. *Polygonum senegalensis*, *Muehlenbeckia tamnifolia* (Kunth) Meisn, *Rheum ribes* L., *Polygonum glabrum*, and other species are validated for antidiabetic potentials [17–20]. Our previous findings suggest that *P. hydropiper* exhibit neuroprotective, gstroprotective, cytotoxic, and antimicrobial properties [21–29]. Based on the antidiabetic potentials of Polygonaceae family and our preliminary studies, we have isolated phytochemicals from the active fraction of *P. hydropiper* and were subjected to enzyme inhibition and molecular docking studies for potential applications in T2DM.

## 2. Materials and Methods

### 2.1. Collection of Plant and Isolation of Phytochemicals

Keeping in view the ethnobotanical importance of Polygonaceae family in diabetes, whole aerial plant of *P. hydropiper* was selected for antidiabetic studies. The plant was collected in 2013 from Talash (Dir KPK), after identification by plant taxonomist Dr. Gul Rahim. A dried sample of the same was secured in the herbarium of University of Malakand, Chakdara (Voucher no. H.UOM.BG.107). The study was approved by Departmental Research Ethics Committee via reference no. DREC/20160502/01. Some initial pilot studies were performed on various fractions of the plant including crude methanolic extract, chloroform, ethyl acetate, *n*-hexane, butanol, and aqueous fractions [30]. Based on preliminary results, chloroform-ethyl acetate fractions were subjected to column chromatography for the isolation of potential compounds. At the start, we observed the elution of phytochemicals from the selected fractions on precoated silica gel TLC plates using increasing polarity elusion systems. Initially, we started the chromatographic process from *n*-hexane only (100%). After some time, we gradually increase the polarity with the addition of 2% ethyl acetate each time (i.e., 98 : 2 < 96 : 4 < 94 : 6 < 92 : 8 < 90 : 10). We visualize the TLC each time and change the polarity accordingly. Thereafter, on the bases of fractions quantity, we selected large-size gravity columns, packed with flash silica slurry and loaded with the selected fractions. Elution was started from nonpolar *n*-hexane towards increase in polarity via addition of ethyl acetate. Partially pure fractions (approximately more than 80%) were collected, run on TLC plates to combined co-elution fractions based on *R*_*f*_ values and were further purified via silica-packed pencil columns. These columns were again carefully eluted with *n*-hexane and chloroform solvent systems for the purification of target compounds. The compounds Ph-1 and Ph-2 were isolated from chloroform fraction, while compounds Ph-3 to Ph-6 were isolated from ethyl acetate bioactive fraction. The compounds Ph-1, Ph-2, Ph-3, Ph-4, Ph-5, and Ph-6 were purified in 250, 275, 122, 145, 270, and 265 mg, respectively.

### 2.2. Structure Elucidation of Isolated Compounds

The purified compounds were subjected to the removal of trace solvents using rotary evaporator. Subsequently, ^1^H NMR analysis was performed to confirm about the compound structure and compared it with the literature. Further, ^13^C NMR analysis was performed to identify the carbon-skeleton, whereas further confirmations were done via mass spectrometry [31, 32].

### 2.3. Antioxidant Studies via DPPH Assay

DPPH antiradicals study was conducted in accordance with our previously published protocol [33, 34]. Initially, 0.1% methanolic-DPPH solution was made and its 100 *µ*L was mixed with same volume (100 *µ*L) of test samples in 96-well plates, followed by incubation for about thirty min in a dark place 25 ± 3°C. Solutions of the provided samples were prepared in concentrations 1000-62.5 *µ*g·mL^−1^. Likewise, Trolox methanolic solution (100 *µ*M) was prepared and subsequently its five concentrations/dilutions 1000, 500, 250, 125, and 62.5 *µ*M were prepared. Subsequent to incubation, a decline in DPPH color was observed and absorbance was recorded at 540 nm using microplate reader [35]. Percent scavenging was estimated as follows:

(1) $$\begin{array}{c} \%\text{ scavenging}=\left[\left(A0-A1\right)/A0\right]\times 100. \end{array}$$

### 2.4. Antioxidant Studies via ABTS Assay

ABTS antiradicals' study was performed following our earlier reported protocol [36, 37]. For preparation of ABTS solution, 192 mg of ABTS salt was mixed in distilled water with subsequent transfer to fifty-milliliter flask and volume adjustments. Thereafter, 1 mL of the previous solution was added to 17 *µ*L of 140 mM potassium persulphate and the mixture was left in the dark for 24 hours. Subsequently, 1 mL of the reaction mixture was diluted to 50 mL using methanol to obtain the final ABTS dilution used in the assay. Consequently, 190-microliter ABTS solution was added to 10 *µ*L sample solutions and in 96-well plates and were incubated for 2 hrs in dark place at room temperature. After the incubation, decline in color intensity of ABTS was measured at 734 nm via microplate reader. Solutions of positive control were prepared in at the concentrations of 1000-62.5 *µ*M. Percent ABTS scavenging was determined using equation:

(2) $$\begin{array}{c} \%\text{ABTS scavenging}=\frac{\text{Control absorbance}-\text{Sample absorbance}}{\text{Control absorbance}}\times 100\text{.} \end{array}$$

### 2.5. *α*-Glucosidase Inhibition Assay

For *α*-glucosidase inhibition assay, 50 *µ*L solution containing 0.5 U·mL^−1^ was added to 50 *µ*L phosphate buffer (6.8 pH) and was added to various concentrations of test samples. The resultant mixture was properly mixed and stored at 37°C for fifteen min. After incubation, 100 *µ*L (3 mM) pNPG solution was added to it. The reaction mixture was again incubated for 10 min at 37°C and finally 0.1 M Na_2_CO_3_ was added to stop reaction [34]. Absorption was measured at 405 nm using 96-well microplate reader. Absorption of mixture without addition of test samples was used as control and without substrate was used as blank. Percent inhibitions were calculated using the formula

(3) $$\begin{array}{c} \%\text{ Inhibition}=\left[1-\left\{\frac{\text{absorbance of sample}-\text{absorbance of blank}}{\text{absorbance of control}}\right\}\right]\times 100. \end{array}$$

### 2.6. Inhibition of *α*-Amylase Enzyme

Using this assay, a previously reported protocol was followed [38]. In brief, 100 *µ*L of 1% starch solution prepared in 29 mM phosphate buffer (pH 6.9) was mixed with 100 *µ*L of sample solution (1000-62.5 *µ*g·mL^−1^) and stored for about 10 min at 25°C in air-tight tubes. Subsequently, 100 *µ*L of 0.5 mg·mL^−1^*α*-amylase solution was added to the mixture and again incubated for 10 min at 25°C. Reaction was stopped via the addition of 200 *µ*L of dinitrosalicylic acid was added and incubated for an additional 5 min in boiling water bath and allowed to cool again. Finally, 50 *µ*L solutions was transferred to 96-well microplate reader and further diluted to 200 *µ*L via the addition of distilled water. Absorbencies were recorded at 540 nm and percent inhibitions were calculated using the equation

(4) $$\begin{array}{c} \%\text{ Inhibition}=\left[1-\left\{\frac{\Delta \text{ A Samples}}{\Delta \text{ A Control}}\right\}\right]\times 100. \end{array}$$

### 2.7. Molecular Docking Studies

*In silico* studies involving the interactions of test drugs with its molecular targets are an important tool in drug discovery [39, 40]. MOE-Dock was used to appraise the binding interactions of our test samples in the active site of *α*-glucosidase and *α*-amylase enzymes. As the *α*-glucosidase enzyme crystal structure is available, so previously reported homology model [41] was used, whereas the 3D crystal structure of *α*-amylase enzyme (4W93) was obtained from Protein Data Bank. Prior to molecular docking, the crystal structures were devoid of water molecules or any ions via Molecular Operating Environment software. Using 3D protonation, hydrogen atoms were added to the proteins and the energy was minimized via MOE parameters (i.e., gradient of 0.05, force field was Amber 99).

Structures of our test samples were initially built using MOE and the energy was reduced. Subsequently, both enzymes were allowed to interact and dock with test samples through MOE preset parameter including placement at Triangle Matcher and rescoring with London dG. Ten confirmations were generated for every compound and top-rank confirmations of the compounds were selected for further analysis. Following molecular docking, the best poses with polar, arene-arene, H-pi, and pi-H interactions were assessed by PyMOL software.

### 2.8. Statistical Analysis

All tests were done three times and results were presented as Mean ± SEM of the collected data. One-way ANOVA coupled with multiple-comparison Dunnett's test was applied for the statistical assessment of the test samples to the control groups.

## 3. Results and Discussion

### 3.1. Identification of the Phytochemicals

**Figure 1:**
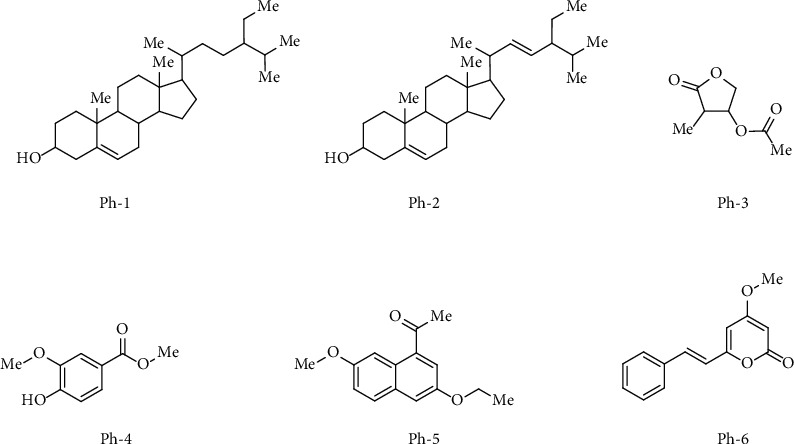
Structures of isolated bioactive compounds from *P. hydropiper*.

In this research, we have isolated six bioactive compounds (Ph-1 to Ph-6) as shown in Figure 1 and File S1. The two compounds (Ph-1 and Ph-2) are steroidal compounds isolated. Compound Ph-3 is a furan derivative. The rest of compounds Ph-4 to Ph-6 are aromatic bioactive compounds. The phytochemicals were identified via ^1^H-NMR, ^13^C-NMR as well as mass-spectrometry assessments. Compounds spectral data is available as supporting information.

The compound Ph-1 was isolated as pure white solid. The molecular weight was confirmed as 414 by mass analysis. The compound Ph-2 was also isolated in white solid form. The observed molecular weight of Ph-2 was 212. In both of these steroidal compounds, majority of the protons are giving splitting in the upfield region due to the presence of aliphatic skeleton. The splitting of protons associated with the double bond or the hydroxy group is showing up in the downfield area. In both steroidal compounds, the methyl groups are majorly showing up signals between chemical shifts 1-2. The methylene and methyne unit are slightly downfield as compared to the methyl groups. The hydroxyl proton in both of the compounds is showing up between chemical shifts 3 and 3.5. Similarly, the alkene protons appeared in the downfield area of 5.2. We have previously reported both Ph-1 and Ph-2 with anti-Alzheimer and anti-cancer activities [37, 42].

The compound Ph-3 is a furan-type derivative isolated as yellowish semisolid form. This derivative contains dominant groups like ester, ketone, and methyl. The compound spectra are shown in the supporting information. The methyl group attached to the carbonyl is giving up a singlet in around 2 chemical shifts. The second methyl group attached to the furan moiety is giving up splitting at upfield area of three protons. Similarly, all the ten protons in the structure are shown in the proton NMR. The compound Ph-4, a phenolic type of derivative, was isolated as brownish oil. The two distinct methoxy groups (each with three protons) appeared slightly upfield to chemical shift 4. Similarly, the phenolic hydroxyl group appeared as a singlet at 5.18 chemical shift. The three aromatic protons appeared in the range of 7.15 to 7.30. The molecular weight of compound Ph-4 was confirmed as 182 by mass analysis as shown in the spectrum in supporting information. The compound Ph-5 was isolated in small amount as turbid semisolid. The ethyl substituent gave two splittings (i.e., a triplet of three protons for methyl group and a quartet of two protons for methylene group). The triplet appeared at 1.08 while the quartet, due to the phenoxy attachment, appeared at 4.63. The methoxy group appeared at 3.95. The methyl group appeared as a singlet at 2.10. The aromatic protons appeared in the downfield region of 7.26 to 7.81. The compound Ph-6 appeared as dominant spot on TLC plate due to the presence of aromatic-conjugated system. The methoxy group with three protons appeared at 3.85. Two singlets (each of one proton) appeared at 5.28 and 5.82. A multiplet of two protons was also observed at 6.50 to 6.60. The phenyl group appeared in the range of 7.24 to 7.50 as multiplets of five protons.

### 3.2. DPPH Antiradicals Assay

**Figure 2:**
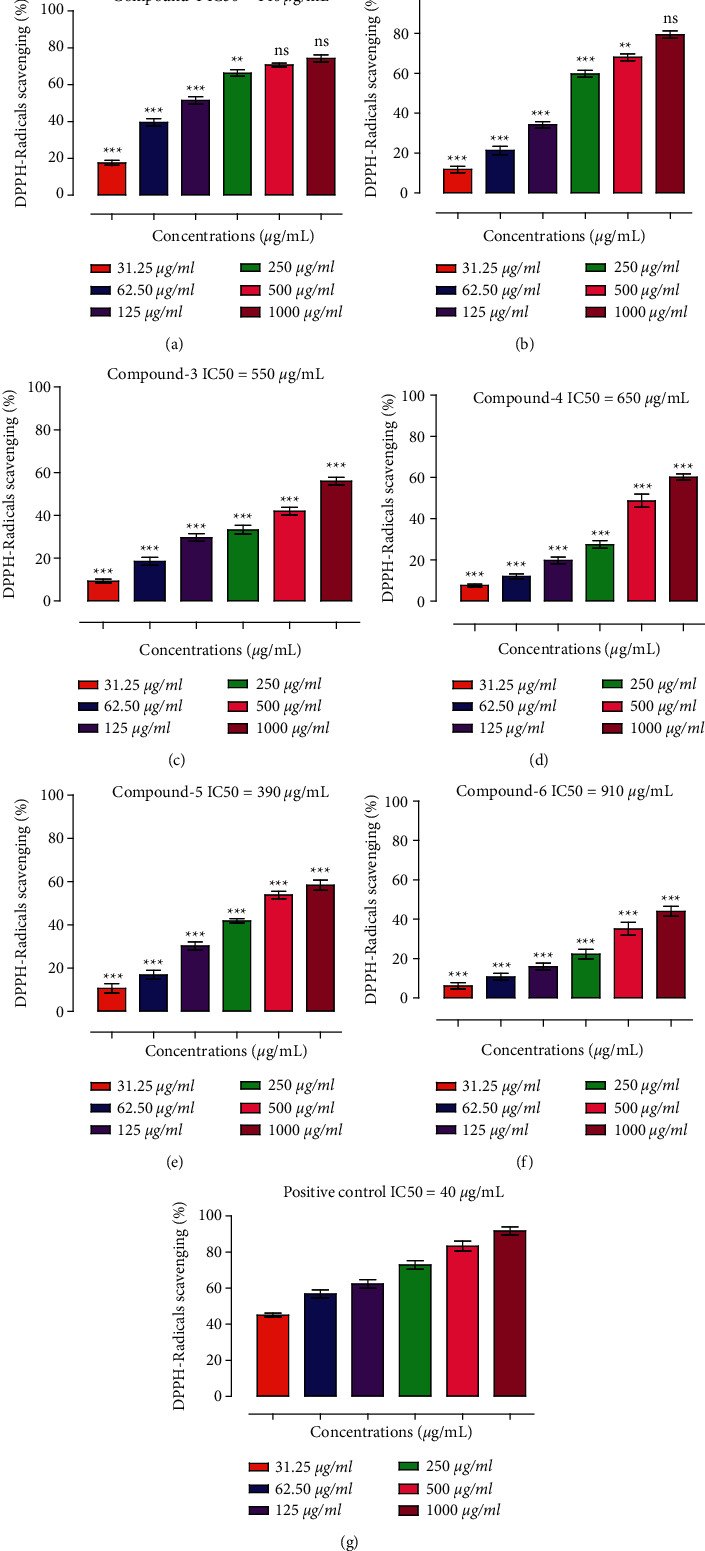
Results of the DPPH antiradicals assay exhibited by isolated compounds. (a) Ph-1, (b) Ph-2, (c) Ph-3, (d) Ph-4, (e) Ph-5, (f) Ph-6, and (g) positive control/ascorbic acid.

In DPPH antiradicals assay, Ph-1 exhibited considerable scavenging effect against DPPH radicals (Figure 2). Ph-1 at concentrations of 31.25, 62.50, 125, 250, 500, and 1000 *µ*g·mL^−1^ showed 17.51, 39.49, 51.38, 66.28, 70.62, and 74.19% scavenging effect, respectively. The IC_50_ value for Ph-1 was 140 *µ*g·mL^−1^. Likewise, percent scavenging potentials of Ph-2 was observed to be 11.83, 21.33, 34.16, 59.66, 68, and 79.33 at the same tested concentrations correspondingly with IC_50_ of 200 *µ*g·mL^−1^. Other compounds were comparatively less potent and exhibited a concentration dependent scavenging effect of 9.2, 18.5, 29.66, 33.33, 42, and 56, respectively, by Ph-3 at the same tested concentrations. Furthermore, Ph-4 showed percent antiradicals potentials of 7.6 (31.25 *µ*g·mL^−1^), 12.06 (62.50 *µ*g·mL^−1^), 19.85 (125 *µ*g·mL^−1^), 27.48 (250 *µ*g·mL^−1^), 48.73 (500 *µ*g·mL^−1^), and 60.26 (1000 *µ*g·mL^−1^) correspondingly, whereas the highest inhibitory activities of Ph-5 and Ph-6 were 58.33 and 44% at 1000 *µ*g·mL^−1^ correspondingly. IC_50_ *µ*g·mL^−1^ for Ph-3 (550 *µ*g·mL^−1^), Ph-4 (650 *µ*g·mL^−1^), Ph-5 (390 *µ*g·mL^−1^), and Ph-6 (910 *µ*g·mL^−1^) was calculated. Positive control (ascorbic acid) showed 45, 56.83, 62.33, 72.85, 83.33, and 91.80% free radicals scavenging potentials at 31.25, 62.50, 125, 250, 500, and 1000 *µ*g·mL^−1^ correspondingly and an IC_50_ of 40 *µ*g·mL^−1^.

### 3.3. ABTS Antiradicals Assay

**Figure 3:**
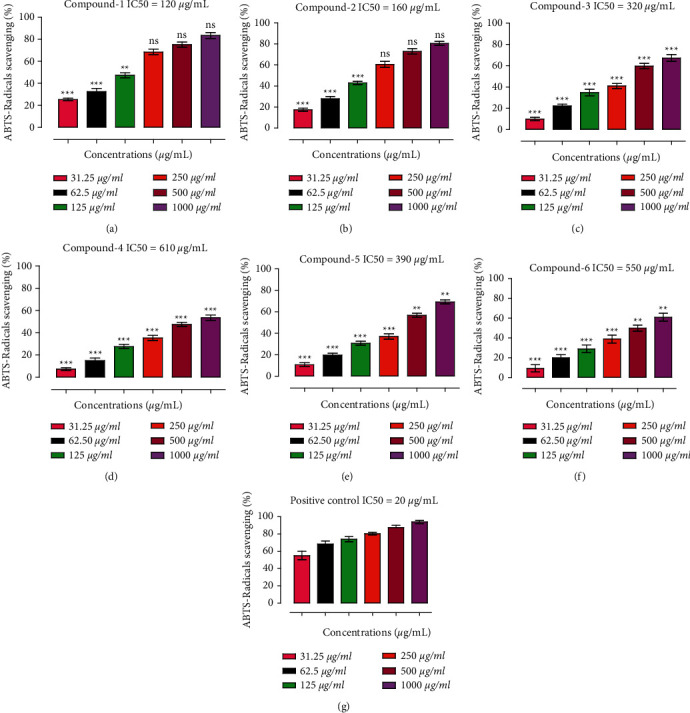
Results of the ABTS antiradicals assay exhibited by isolated compounds. (a) Ph-1, (b) Ph-2, (c) Ph-3, (d) Ph-4, (e) Ph-5, (f) Ph-6, and (g) positive control/ascorbic acid.

In this assay, Ph-1 exhibited considerable free radicals scavenging effect. Ph-1 at the tested concentrations of 1000, 500, 250, 125, 62.5, and 31.25 *µ*g·mL^−1^ showed 83.44, 75.22, 68.47, 47.34, 32.58, and 25.25% inhibitions, respectively, against ABTS radicals. The IC_50_ value was 120 *µ*g·mL^−1^ at the tested concentrations. Likewise, Ph-2 was comparably effective against ABTS radicals with percent inhibitions of 80.63, 72.68, 60.56, 42.89, 27.88, and 17.44, respectively, at the same concentrations. The IC_50_ of Ph-2 against ABTS radicals was 160 *µ*g·mL^1^. The percent inhibitions of Ph-1 and Ph-2 were very comparable with the positive control which showed 94% inhibition at the highest tested concentration (1000 *µ*g·mL^−1^) and an IC_50_ of 20 *µ*g·mL^−1^ (Figure 3). Other compounds also displayed concentration-dependent moderate antiradicals potentials. Ph-3 showed 67.16% inhibition at the highest tested concentrations (1000 *µ*g·mL^−1^) with IC_50_ of 320 *µ*g·mL^−1^. Furthermore, Ph-4 (53.66%), Ph-5 (69.16%), and Ph-6 (61%) inhibitions were observed at 1000 *µ*g·mL^−1^, respectively, whereas the lowest concentrations were less effective (Figure 3). The IC_50_ values for Ph-4 (610 *µ*g·mL^−1^), Ph-5 (350 *µ*g·mL^−1^), and Ph-6 (550 *µ*g·mL^−1^).

### 3.4. *α*-Glucosidase Inhibition Study

Results of *α*-glucosidase inhibition studies are summarized in Table 1. All isolated compounds exhibited concentration-dependent inhibition against *α*-glucosidase enzyme. Compounds Ph-1 and Ph-2 were most potent with 81.84 ± 0.32 and 78.79 ± 0.63% enzyme inhibitions at 1000 *µ*g·mL^−1^, respectively. Ph-1 and Ph-2 exhibited IC_50_s of 85 and 170 *µ*g·mL^−1^ correspondingly. Inhibitory results of both compounds were very comparable with positive control group. Among the other samples, Ph-3, Ph-4, Ph-5, and Ph-6 exhibited 55.44 ± 0.09, 64.36 ± 0.61, 67.37 ± 0.68, and 58. 91 ± 1.54% enzyme inhibition at maximum used concentration of 1000 *µ*g·mL^−1^, respectively. The IC_50_ values for Ph-3, Ph-4, Ph-5, and Ph-6 were 474.83, 455.94, 228, and 337.94 *µ*g·mL^−1^, respectively.

### 3.5. Inhibitory Study against *α*-Amylase

In this study, Ph-1 and Ph-2 exhibited considerable enzyme inhibitory potentials in comparison to standard drug. Percent enzyme inhibitions observed for Ph-1 were 75.83, 69.54, 64.36, 55.84, and 48.8 at the tested concentrations of 1000, 500, 250, 125, and 62.50 *µ*g·mL^−1^, respectively. Likewise, Ph-2 displayed 71.62, 63.86, 44.48, 37.54, and 31.74% inhibitions at the same tested concentrations, respectively. IC_50_s for Ph-1, Ph-2 were 69.48 and 252.01 *µ*g·mL^−1^ correspondingly as shown in Table 1. Positive control showed 85.72% enzyme inhibition and IC_50_ of 45 *µ*g·mL^−1^. Among the other compounds, Ph-3, Ph-4, Ph-5, and Ph-6 exhibited 57.33, 72.51, 64.23, and 61.33% inhibitions, respectively, at 1000 *µ*g·mL^−1^. The IC_50_ values for these compounds were Ph-3 (332.19 *µ*g·mL^−1^), Ph-4 (333.25 *µ*g·mL^−1^), Ph-5 (370 *µ*g·mL^−1^), and Ph-6 (271.24 *µ*g·mL^−1^), respectively.

Inhibition of *α*-glucosidase and *α*-amylase is among the most useful strategies to reduce postprandial hyperglycemia in T2DM patients. Natural products are proved to be among the vital sources for drug discovery against DM. Numerous plants have been proved to act as antihyperglycemic agents alone or as complementary therapies with other antidiabetic agents. Medicinal plants and their fruits/seeds including cinnamon, aloe, gymnema, bitter melon, coffee, guava, cocoa, green tea, nettle, garlic, sage, caper, soybean, turmeric, yerba mate, and walnut have got tremendous focus and have scientifically proved antidiabetic potentials. Several isolated compounds from natural origin including phenolics, galegine, pycnogenol, miglitol, voglibose, and acarbose are extensively employed for the management of T2DM [12]. In the current study, Ph-1 and Ph-2 exhibited considerable enzyme inhibition potentials and need further detailed studies in animal models.

### 3.6. Docking Analysis with *α*-Glucosidase Enzyme

**Figure 4:**
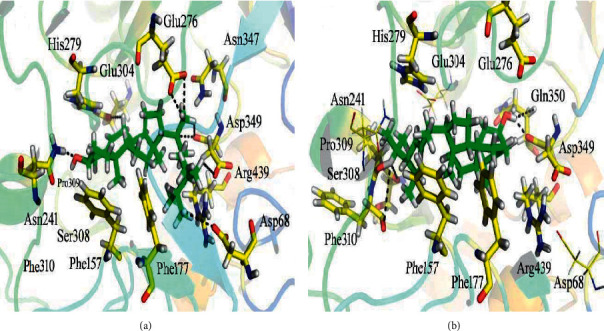
Results of the docking studies of Ph-1 (a) and Ph-2 (b) against *α*-glucosidase enzyme.

As obvious from the results in Table 2, our test samples exhibited considerably good interactions with the target enzymes as indicated by binding energies and binding mode. Our docking results revealed that majority of the compounds displayed top-ranked confirmations with *α*-glucosidase enzyme and majority of the samples were properly accommodated in the enzyme active site as summarized in Table 2. Docking studies displayed best binding with compound Ph-1 (docking score = −12.4286) formed eight hydrogen bonds and three H-pi linkages with the Asp 68, Phe 157, Phe 177, Asn 241, Glu 276, His 279, Phe 300, Glu 304, Ser 308, Pro 309, Phe 310, Asp 349, and Arg 439 residues of the binding pocket of the *α*-glucosidase as shown in Figure 4(a). Asp 68, Glu 276, Asp 349, and Arg 439 formed polar bonds with the 3-ethyl-2-methylpentane moiety of the ligand. Asn 241 made hydrogen bond with the hydroxyl (-OH) moiety while Glu 304 formed H-donor interaction with the –CH group of the 4a,6-dimethyl-1,2,3,4,4a,5,6,7-octahydronaphthalen-2-ol moiety of the inhibitor. Phe 157, Phe 177, and His 279 were observed making H-pi contacts with the ligand as shown in Figure 4(a). We hypothesize that the potency of the ligand can be attributed to the occurrence of the electron-donating group (-OH) along with the electron-cloud system of the compound.

We found Ph-2 as another highly potential compound with docking score of −11.9020. The compound showed five polar interactions with the active residues of the target enzyme (Figure 4(b)). It was observed that Asp 349 and Gln 350 established H-donor and H-acceptor interactions with the hydroxyl (-OH) moiety of the inhibitor, respectively. Ser 308, Pro 309, and Phe 310 formed H-donor interactions with the carbon atom of the 2,3-dimethylpentane moiety whereas Phe 157 and His 279 formed H-pi linkages with the 6-ring moiety of the ligand. The potency of the ligand might be due to the presence of the electron donating group (-OH) as well as delocalized electrons of the ligand.

All the compounds of the series showed good *in silico* results in comparison to positive control (docking score = −5.3602). The control, acarbose, makes seven hydrogen bonds with the active site residues (Table 2). The strength of hydrogen bonds made by acarbose is relatively strong as compared to other compounds, but due to its bulky size it has some clashes with catalytic residues. Therefore, these clashes reduce its docking score when compared with other molecules in the series (Table 2).

### 3.7. Docking Analysis with *α*-Amylase Enzyme

**Figure 5:**
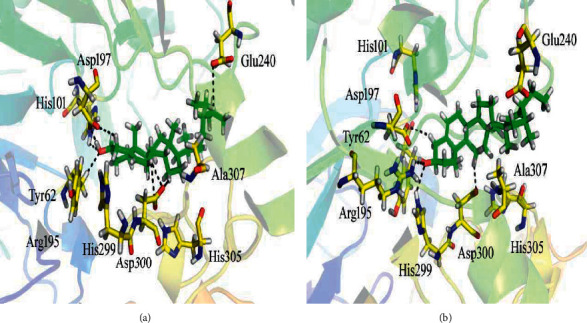
Docking conformation of (a) compound Ph-1 and (b) compound Ph-2 in the active site of *α*-amylase.

Our docking results against *α*-amylase enzyme revealed that majority of them were well accommodated inside the active site residues (Tyr 62, His 101, Arg 195, Asp 197, Glu 240, His 299, Asp 300, His 305, and Ala 307). Ph-1 exhibited docking score of -6.9473 and the test compound produced eight H-bonds at the enzyme active sites.  Figure 5(a) demonstrates the binding mode of the compound *Ph-1*, showing that the hydroxyl group interacts with His 101 and Tyr 62 and forms H-acceptor contacts while with the Asp 197 residue a H-donor interaction was observed. Asp 197 showed H-donor interaction with the carbon atom of the cyclohexanol moiety of the ligand. The carbon atoms of the 1-(5-ethyl-6-methylheptan-2-yl)-7a-methyloctahydro-1H-indene moiety of the inhibitor contacts with the Glu 240 and Asp 300 via hydrogen bonding. Inhibitory potential of the compound can be attributed to the presence of electron donating group (-OH) as well the electronic-cloud system might be implicated in the high enzyme inhibitory property of the test compound.

Among the series of six isolated compounds, the second one good inhibitor was compound Ph-2 (docking score = −6.6032) according to the docking scores (Table 3). The compound Ph-2 showed four polar interactions with the active residues of the *α*-amylase drug target as shown in Figure 5(b). Arg 195 and His 299 form H-acceptor bonds with the hydroxyl moiety of the compound while the carbon atoms of the 4a,6-dimethyl-1,2,3,4,4a,5,6,7-octahydronaphthalen-2-ol moiety of the compound form carbon-based H-donor contacts with the active residues Asp 197 and Asp 300 of the enzyme. Majority of the tested compounds displayed significant docking scores and good interactions at the active sites of the drug target as compared to the standard inhibitor (Montbretin-A) of the *α*-amylase.

## 4. Conclusions

In an activity-guided isolation process, six compounds were isolated from the most active fractions of *P. hydropiper* followed by identification via different techniques. The compounds were tested using antidiabetic and antioxidant studies. All tested compounds exhibited considerable antioxidant (DPPH/ABTS) and enzymes inhibition potentials. Among the tested compounds, Ph-1 and Ph-2 showed highest activity against free radicals and target enzymes. Antioxidant and *α*-glucosidase as well as *α*-amylase enzymes might be important in the antidiabetic potentials of the samples. The binding affinities of the test samples were further confirmed by their interactions at the active sites of the enzyme and low binding energies during the molecular docking studies.

## Figures and Tables

**Table 1:** Results of *α*-glucosidase/*α*-amylase inhibitory assays.

Samples	*α*-Glucosidase inhibition assay			*α*-Amylase Inhibition assay		
	Conc. *µ*g·mL^−1^	% enzyme inhibition	IC_50_*µ*g·mL^−1^	Conc. *µ*g·mL^−1^	% enzyme inhibition	IC_50_*µ*g·mL^−1^
Ph-1	1000 500 250 125 62.50	81.84 ± 0.32^ns^ 75.68 ± 0.22 ^ns^ 67.54 ± 0.16 ^ns^ 52.83 ± 1.07 ^ns^ 39.54 ± 0.46^*∗*^	85	1000 500 250 125 62.50	75.83 ± 1.07 ^ns^ 69.54 ± 0.46 ^ns^ 64.36 ± 0.21 ^ns^ 55.84 ± 0.32 ^ns^ 48.68 ± 0.22 ^ns^	69.48
Ph-2	1000 500 250 125 62.50	78.79 ± 0.63 ^ns^ 70.67 ± 0.61 ^ns^ 51.69 ± 0.77^*∗∗*^ 44.54 ± 0.50^*∗∗*^ 24.00 ± 0.30^*∗∗∗*^	170	1000 500 250 125 62.50	71.62 ± 0.74 ^ns^ 63.86 ± 0.60 ^ns^ 44.48 ± 0.64^*∗∗*^ 37.54 ± 0.50^*∗∗*^ 31.74 ± 0.61^*∗∗∗*^	252.01
Ph-3	1000 500 250 125 62.50	55. 44 ± 0.09^*∗∗∗*^ 51. 10 ± 0.33^*∗∗∗*^ 45. 93 ± 0.45^*∗∗∗*^ 41. 64 ± 0.13^*∗∗∗*^ 34. 00 ± 0.31^*∗∗∗*^	474.83	1000 500 250 125 62.50	57.33 ± 0.88^*∗∗∗*^ 53.00 ± 0.57^*∗∗∗*^ 48.96 ± 1.91^*∗∗∗*^ 43.00 ± 1.73^*∗∗∗*^ 37.66 ± 1.36^*∗∗∗*^	332.19
Ph-4	1000 500 250 125 62.50	64.36 ± 0.61^*∗∗∗*^ 53.40 ± 0.52^*∗∗∗*^ 39.46 ± 0.60^*∗∗∗*^ 22.52 ± 0.49^*∗∗∗*^ 17.52 ± 0.71^*∗∗∗*^	455.94	1000 500 250 125 62.50	72.51 ± 0.62^ns^ 63.44 ± 0.44 ^ns^ 42.46 ± 0.47^*∗∗*^ 23.68 ± 0.64^*∗∗∗*^ 19.31 ± 0.57^*∗∗∗*^	333.25
Ph-5	1000 500 250 125 62.50	67.37 ± 0.68 61.58 ± 0.74 56.65 ± 0.77 47.55 ± 0.77 43.46 ± 0.63	228	1000 500 250 125 62.50	64.23 ± 0.44^*∗∗∗*^ 57.45 ± 0.65^*∗∗∗*^ 52.37 ± 0.64^*∗∗∗*^ 47.37 ± 0.54^*∗∗∗*^ 42.30 ± 0.61^*∗∗*^	370
Ph-6	1000 500 250 125 62.50	58. 91 ± 1.54^*∗∗∗*^ 54. 49 ± 0.14^*∗∗*^ 48. 89 ± 0.20^*∗∗*^ 41. 43 ± 0.29^*∗∗*^ 37.23 ± 0.44^*∗∗*^	337.94	1000 500 250 125 62.50	61.33 ± 0.88^*∗∗∗*^ 55.00 ± 0.57^*∗∗∗*^ 50.96 ± 1.91^*∗∗∗*^ 43.00 ± 1.73^*∗∗∗*^ 36.09 ± 0.32^*∗∗∗*^	271.24
P. Control	1000 500 250 125 62.50	89.42 ± 0.55 77.52 ± 0.62 71. 36 ± 0.49 62.39 ± 0.49 55.47 ± 0.52	40	1000 500 250 125 62.50	85.72 ± 0.66 78.60 ± 0.46 73.62 ± 0.40 65.48 ± 0.74 59.36 ± 0.57	45

For the statistical difference among the test samples and positive control, one-way ANOVA followed by multiple comparison Dunnett's test was applied to the data. ^*∗∗∗*^, *p* < 0.001, ^*∗∗*^*p* < 0.01, *p* < 0.01 and ns: values not significantly different when compared with standard drug inhibitions (Acarbose) at the same tested concentrations.

**Table 2:** Docking scores and report of predicted interactions of docked conformations of compounds against *α*-glucosidase.

S. no.	Ligand		Receptor			Interaction	Distance	E (kcal/mol)	Docking score
1	C	16	OE2	GLU	304	H-donor	3.38	−0.1	−12.4286
	C	40	O	ASP	349	H-donor	2.93	−0.1	
	C	42	OD2	ASP	349	H-donor	3.88	−0.1	
	C	45	OE1	GLU	276	H-donor	2.79	−0.1	
	C	45	OE2	GLU	276	H-donor	3.54	−0.1	
	C	52	OD2	ASP	349	H-donor	3.9	−0.1	
	C	56	OD2	ASP	68	H-donor	3.17	−0.1	
	O	79	ND2	ASN	241	H-acceptor	2.89	−3	
	C	6	5-ring	HIS	279	H-pi	4.28	−0.1	
	C	11	6-ring	PHE	157	H-pi	3.74	−0.9	
	C	54	6-ring	PHE	177	H-pi	3.75	−0.3	
2	C	57	O	SER	308	H-donor	4.13	−0.1	−11.9020
	C	57	O	PRO	309	H-donor	3.53	−0.1	
	C	57	O	PHE	310	H-donor	3.43	−0.1	
	O	69	O	ASP	349	H-donor	2.85	−1.5	
	O	69	CG	GLN	350	H-acceptor	3.33	−0.1	
	C	44	6-ring	PHE	157	H-pi	3.15	−0.1	
	C	48	5-ring	HIS	279	H-pi	4.54	−0.1	
	C	61	5-ring	HIS	279	H-pi	4.52	−0.2	
3	C	3	O	ASP	349	H-donor	3.03	−1	−7.4657
	O	10	CG2	VAL	303	H-acceptor	3.87	−0.1	
	O	17	CE1	TYR	313	H-acceptor	2.93	−0.4	
4	C	13	OE1	GLN	350	H-donor	3.29	−0.1	−6.8211
	C	13	6-ring	PHE	300	H-pi	4.73	−0.3	
	−6	ring	CD	ARG	312	pi-H	4.2	−0.1	
5	C	17	OD2	ASP	68	H-donor	3.8	−0.1	−10.6611
	C	25	O	ASP	349	H-donor	3.84	−0.1	
	C	25	OE1	GLN	350	H-donor	3.66	−0.1	
	O	30	NE2	HIS	348	H-acceptor	2.71	−2	
	C	13	5-ring	HIS	348	H-pi	4.48	−0.4	
	−6	ring	CD	ARG	439	pi-H	4.15	−0.1	
6	C	3	O	SER	156	H-donor	3.63	−0.1	−10.7079
	C	6	OD2	ASP	408	H-donor	3.58	−0.1	
	C	27	OE1	GLN	350	H-donor	3.65	−0.2	
	C	6	6-ring	PHE	311	H-pi	3.67	−0.1	
	−6-ring		CB	ARG	312	pi-H	4.91	−0.2	
Acarbose	C	23	OD1	ASP	214	H-donor	2.91	−0.8	−5.3602
	O	33	OE1	GLN	181	H-donor	3.25	−1	
	O	34	OD2	ASP	408	H-donor	3.57	−0.5	
	O	37	OD2	ASP	214	H-donor	2.86	−2.5	
	O	16	ND2	ASN	347	H-acceptor	2.7	−3	
	O	25	NE	ARG	312	H-acceptor	2.56	−3.5	
	O	37	NE2	HIS	111	H-acceptor	2.49	−2.7	
	N	32	6-ring	PHE	177	Cation-pi	4.04	−1	

**Table 3:** Docking scores and report of predicted interactions of docked conformations of compounds against *α*-amylase.

S. no.	Ligand		Receptor				Interaction	Distance	E (kcal/mol)	Docking score
1	C	6	OD1	ASP	197	(A)	H-donor	3.38	−0.1	−6.9473
	C	16	OD1	ASP	300	(A)	H-donor	3.54	−0.1	
	C	16	OD2	ASP	300	(A)	H-donor	3.42	−0.1	
	C	32	OD2	ASP	300	(A)	H-donor	3.64	−0.1	
	C	59	OE2	GLU	240	(A)	H-donor	3.59	−0.1	
	O	79	OD2	ASP	197	(A)	H-donor	2.56	−1.9	
	O	79	CB	TYR	62	(A)	H-acceptor	3.36	−0.3	
	O	79	NE2	HIS	101	(A)	H-acceptor	2.98	−1	
2	C	6	OD2	ASP	197	(A)	H-donor	3.93	−0.1	−6.6032
	C	11	OD1	ASP	300	(A)	H-donor	3.44	−0.1	
	O	69	NH2	ARG	195	(A)	H-acceptor	3.23	−0.3	
	O	69	NE2	HIS	299	(A)	H-acceptor	3.45	−0.2	
3	O	10	CZ2	TRP	58	(A)	H-acceptor	3.38	−0.1	−4.2675
4	C	5	OD2	ASP	197	(A)	H-donor	3.33	−0.1	−4.3894
	O	19	NH2	ARG	195	(A)	H-acceptor	2.88	−1.7	
	O	19	NE2	HIS	299	(A)	H-acceptor	2.93	−4.4	
	6-ring		CB	ALA	198	(A)	pi-H	3.73	−0.4	
5	C	13	OD1	ASP	300	(A)	H-donor	2.94	−0.2	58.5320
6	C	6	O	THR	163	(A)	H-donor	3.71	−0.1	−4.0766
	O	25	CD2	LEU	162	(A)	H-acceptor	3.84	−0.1	
	6-ring		CD1	LEU	162	(A)	pi-H	4.31	−0.2	
P.C	O3F	7	OD2	ASP	300	(A)	H-donor	2.59	−2.5	−4.0028
	O29	55	O	TYR	62	(A)	H-donor	2.65	−3.2	
	O2A	70	OE2	GLU	240	(A)	H-donor	2.72	−3.3	
	O3A	72	OE1	GLU	240	(A)	H-donor	2.75	−3.4	
	O9F	16	NE2	HIS	305	(A)	H-acceptor	2.92	−4.1	

P.C: positive control/standard.

## Data Availability

Data will be available from the corresponding authors upon request.

## Supplementary Materials

Supplementary Materials Supplementary file S1 contains spectral details of the isolated compounds.

## References

[1] Sadiq A., Rashid U., Ahmad S. (2020). Treating hyperglycemia from eryngium caeruleum M. Bieb: in-vitro *α*-glucosidase, antioxidant, in-vivo antidiabetic and molecular docking-based approaches. *Frontiers of Chemistry*.

[2] King H., Aubert R. E., Herman W. H. (1998). Global burden of diabetes, 1995–2025: prevalence, numerical estimates, and projections. *Diabetes Care*.

[3] Nair S. S., Kavrekar V., Mishra A. (2013). In vitro studies on alpha amylase and alpha glucosidase inhibitory activities of selected plant extracts. *European Journal of Experimental Biology*.

[4] Ahmad A., Ullah F., Sadiq A. (2020). Comparative cholinesterase, *α*-glucosidase inhibitory, antioxidant, molecular docking, and kinetic studies on potent succinimide derivatives. *Drug Design, Development and Therapy*.

[5] Liu L., Deseo M. A., Morris C., Winter K. M., Leach D. N. (2011). Investigation of *α*-glucosidase inhibitory activity of wheat bran and germ. *Food Chemistry*.

[6] Ortiz-Andrade R. R., García-Jiménez S., Castillo-España P., Ramírez-Ávila G., Villalobos-Molina R., Estrada-Soto S. (2007). *α*-Glucosidase inhibitory activity of the methanolic extract from *Tournefortia hartwegiana*: an anti-hyperglycemic agent. *Journal of Ethnopharmacology*.

[7] Matsui T., Ueda T., Oki T., Sugita K., Terahara N., Matsumoto K. (2001). *α*-Glucosidase inhibitory action of natural acylated anthocyanins 1 survey of natural pigments with potent inhibitory activity. *Journal of Agricultural and Food Chemistry*.

[8] Bedekar A., Shah K., Koffas M. (2010). Natural products for type II diabetes treatment. *Advances in Applied Microbiology*.

[9] Perla V., Jayanty S. S. (2013). Biguanide related compounds in traditional antidiabetic functional foods. *Food Chemistry*.

[10] Schäfer A., Högger P. (2007). Oligomeric procyanidins of French maritime pine bark extract (Pycnogenol®) effectively inhibit *α*-glucosidase. *Diabetes Research and Clinical Practice*.

[11] Kim Y.-M., Jeong Y.-K., Wang M.-H., Lee W.-Y., Rhee H.-I. (2005). Inhibitory effect of pine extract on *α*-glucosidase activity and postprandial hyperglycemia. *Nutrition*.

[12] Ríos J. L., Francini F., Schinella G. R. (2015). Natural products for the treatment of type 2 diabetes mellitus. *Planta Medica*.

[13] Ayaz M., Junaid M., Ahmed J. (2014). Phenolic contents, antioxidant and anticholinesterase potentials of crude extract, subsequent fractions and crude saponins from *Polygonum hydropiper* L. *BMC Complementary and Alternative Medicine*.

[14] Angothu S., Lakshmi S. M., Kumar A. S., Reddy K. Y. (2010). Antidiabetic activity of aerial parts of Antigonon leptopus Hook & Arn in alloxan-induced diabetic rats. *International Journal of Phytopharmacology*.

[15] Raghavendra H. (2018). Medicinal uses, phytochemistry and pharmacological activities of Antigonon leptopus Hook. and Arn.(Polygonaceae): a review. *Journal of Chemical and Pharmaceutical Research*.

[16] Oany A. R., Hossain M. U., Islam R., Emran A.-A. (2017). A preliminary evaluation of cytotoxicity, antihyperglycemic and antinociceptive activity of *Polygonum hydropiper* L. ethanolic leaf extract. *Clinical Phytoscience*.

[17] Yildirim M., Degirmenci U., Akkapulu M. (2021). The effect of Rheum ribes L. on oxidative stress in diabetic rats. *Journal of Basic and Clinical Physiology and Pharmacology*.

[18] Torres-Naranjo M., Suárez A., Gilardoni G., Cartuche L., Flores P., Morocho V. (2016). Chemical constituents of *Muehlenbeckia tamnifolia* (kunth) Meisn (Polygonaceae) and its in vitro *α*-amilase and *α*-glucosidase inhibitory activities. *Molecules*.

[19] Bothon F. T., Debiton E., Avlessi F., Forestier C., Teulade J. C., Sohounhloue D. K. (2013). In vitro biological effects of two anti-diabetic medicinal plants used in Benin as folk medicine. *BMC Complementary and Alternative Medicine*.

[20] Lo V. N. (2005). Materia medica with hypoglycemia and anti-diabetic effects. *Pharmaceutical Journal*.

[21] Tong X., Li X., Ayaz M. (2020). Neuroprotective studies on *Polygonum hydropiper* L. essential oils using transgenic animal models. *Frontiers in Pharmacology*.

[22] Ayaz M., Ullah F., Sadiq A., Kim M. O., Ali T. (2019). Editorial: natural products-based drugs: potential therapeutics against alzheimer’s disease and other neurological disorders. *Frontiers in Pharmacology*.

[23] Ayaz M., Junaid M., Ullah F. (2016). Molecularly characterized solvent extracts and saponins from Polygonum hydropiper L. show high anti-angiogenic, anti-tumor, brine shrimp, and fibroblast NIH/3T3 cell line cytotoxicity. *Frontiers in Pharmacology*.

[24] Ayaz M., Junaid M., Ullah F. (2017). GC-MS analysis and gastroprotective evaluations of crude extracts, isolated saponins, and essential oil from *Polygonum hydropiper* L. *Frontiers of Chemistry*.

[25] Ayaz M., Junaid M., Ullah F. (2016). Chemical profiling, antimicrobial and insecticidal evaluations of *Polygonum hydropiper* L. *BMC Complementary and Alternative Medicine*.

[26] Ayaz M., Junaid M., Subhan F. (2014). Heavy metals analysis, phytochemical, phytotoxic and anthelmintic investigations of crude methanolic extract, subsequent fractions and crude saponins from *Polygonum hydropiper* L. *BMC Complementary and Alternative Medicine*.

[27] Ayaz M., Ahmad I., Sadiq A. (2020). *Persicaria hydropiper* (L.) Delarbre: a review on traditional uses, bioactive chemical constituents and pharmacological and toxicological activities. *Journal of Ethnopharmacology*.

[28] Ayaz M., Junaid M., Ullah F. (2015). Comparative chemical profiling, cholinesterase inhibitions and anti-radicals properties of essential oils from Polygonum hydropiper L: a Preliminary anti- Alzheimer’s study. *Lipids in Health and Disease*.

[29] Mahnashi M. H., Alqahtani Y. S., Alyami B. A. (2021). Cytotoxicity, anti-angiogenic, anti-tumor and molecular docking studies on phytochemicals isolated from Polygonum hydropiper L. *BMC Complementary Medicine and Therapies*.

[30] Zeb A., Ahmad S., Ullah F., Ayaz M., Sadiq A. (2016). Anti-nociceptive activity of ethnomedicinally important analgesic plant isodon rugosus wall. ex benth: mechanistic study and identifications of bioactive compounds. *Frontiers in Pharmacology*.

[31] Ahmad S., Mahnashi M. H., Alyami B. A. (2021). Synthesis of michael adducts as key building blocks for potential analgesic drugs: in vitro, in vivo and in silico explorations. *Drug Design, Development and Therapy*.

[32] Majid Shah S., Ullah F., Ayaz M. (2019). *β*-Sitosterol from Ifloga spicata (Forssk.) Sch. Bip. as potential anti-leishmanial agent against leishmania tropica: docking and molecular insights. *Steroids*.

[33] Boly R., Lamkami T., Lompo M., Dubois J., Guissou I. (2016). DPPH free radical scavenging activity of two extracts from agelanthus dodoneifolius (Loranthaceae) leaves. *International Journal of Toxicological and Pharmacological Research*.

[34] Ovais M., Ayaz M., Khalil A. T. (2018). HPLC-DAD finger printing, antioxidant, cholinesterase, and *α*-glucosidase inhibitory potentials of a novel plant Olax nana. *BMC Complementary and Alternative Medicine*.

[35] Chen Z., Bertin R., Froldi G. (2013). EC50 estimation of antioxidant activity in DPPH assay using several statistical programs. *Food Chemistry*.

[36] Mir N. T., Saleem U., Anwar F. (2019). Lawsonia Inermis markedly improves cognitive functions in animal models and modulate oxidative stress markers in the brain. *Medicina*.

[37] Ayaz M., Junaid M., Ullah F. (2017). Anti-Alzheimer’s studies on *β*-sitosterol isolated from *Polygonum hydropiper* L. *Frontiers in Pharmacology*.

[38] Karakaya S., Gözcü S., Güvenalp Z. (2018). The *α*-amylase and *α*-glucosidase inhibitory activities of the dichloromethane extracts and constituents of ferulago bracteata roots. *Pharmaceutical Biology*.

[39] Leach A. R., Shoichet B. K., Peishoff C. E. (2006). Prediction of protein-ligand interactions. docking and scoring: successes and gaps. *Journal of Medicinal Chemistry*.

[40] Ghufran M., Rehman A. U., Shah M., Ayaz M., Ng H. L., Wadood A. (2020). In-silico design of peptide inhibitors of K-ras target in cancer disease. *Journal of Biomolecular Structure and Dynamics*.

[41] Liu M., Zhang W., Wei J., Lin X. (2011). Synthesis and *α*-glucosidase inhibitory mechanisms of bis (2,3-dibromo-4,5-dihydroxybenzyl) ether, a potential marine bromophenol *α*-glucosidase inhibitor. *Marine Drugs*.

[42] Ayaz M., Sadiq A., Wadood A., Junaid M., Ullah F., Zaman Khan N. (2019). Cytotoxicity and molecular docking studies on phytosterols isolated from *Polygonum hydropiper* L. *Steroids*.

