# Supplementary material for: Antioxidant, Enzyme Inhibitory, and Molecular Docking Approaches to the Antidiabetic Potentials of Bioactive Compounds from Persicaria hydropiper L
Source: Evid Based Complement Alternat Med. 2022 Apr 14;2022:6705810. doi: 10.1155/2022/6705810 (PMC9023165; doi:10.1155/2022/6705810)
Supplement: Supplementary Materials — Supplementary file S1 contains spectral details of the isolated compounds. [file 6705810.f1.docx]

**Anti-oxidant, Enzyme inhibitory and molecular docking approaches to the anti-diabetic potentials of bioactive compounds from *Persicaria hydropiper* L.**

Muhammad Ayaz^1^*, Abdul Sadiq^1^, Osama F. Mosa^2^, Tariq Abdalla Zafar^2^, Alashary Adam Eisa Hamdoon^2^, Modawy Alnour Khalifa^2^, Mohamed Ahmed Elawad^2^, Alshebli Abdelnabi Sharaf El-dein^2^, Farhat Ullah^1^, Mehreen Ghufran^3^, Atul Kabra^4^*

^1^Department of Pharmacy, Faculty of Biological Sciences, University of Malakand, Chakdara, 18000 Dir (L), KP, Pakistan. [ayazuop@gmail.com](mailto:ayazuop@gmail.com); [sadiquom@yahoo.com](mailto:sadiquom@yahoo.com); [farhataziz80@hotmail.com](mailto:farhataziz80@hotmail.com)

^2^Public health Department Health Sciences College at Lieth, Umm Al Qura University, Makkah, KSA. [drosama.moussa@gmail.com](mailto:drosama.moussa@gmail.com); [tariqzafar_79@hotmail.com](mailto:tariqzafar_79@hotmail.com); [alashary1226@gmail.com](mailto:alashary1226@gmail.com); [memkhalifa@uqu.edu.sa](mailto:memkhalifa@uqu.edu.sa)**;** [wadelawad32@yahoo.com](mailto:wadelawad32@yahoo.com); [aahmed@uqu.edu.sa](mailto:aahmed@uqu.edu.sa)

^3^Department of Biochemistry, UCS, Shankar, Abdul Wali Khan University, Mardan, Mardan-23200, Pakistan. [mehreen@awkum.edu.pk](mailto:mehreen@awkum.edu.pk)

^4^University Institute of Pharma Sciences, Chandigarh University, Gharuan, Mohali-140413, Punjab, India. [atul.kbr@gmail.com](mailto:atul.kbr@gmail.com)

**Correspondence:**

(Atul Kabra) [atul.kbr@gmail.com](mailto:atul.kbr@gmail.com); (Muhammad Ayaz) [ayazuop@gmail.com](mailto:ayazuop@gmail.com)

**Compound Ph-1**

**17-(5-ethyl-6-methylheptan-2-yl)-10,13-dimethyl-2,3,4,7,8,9,10,11,12,13,14,15,16,17-tetradecahydro-1H-cyclopenta[a]phenanthren-3-ol (Ph-1)**

####
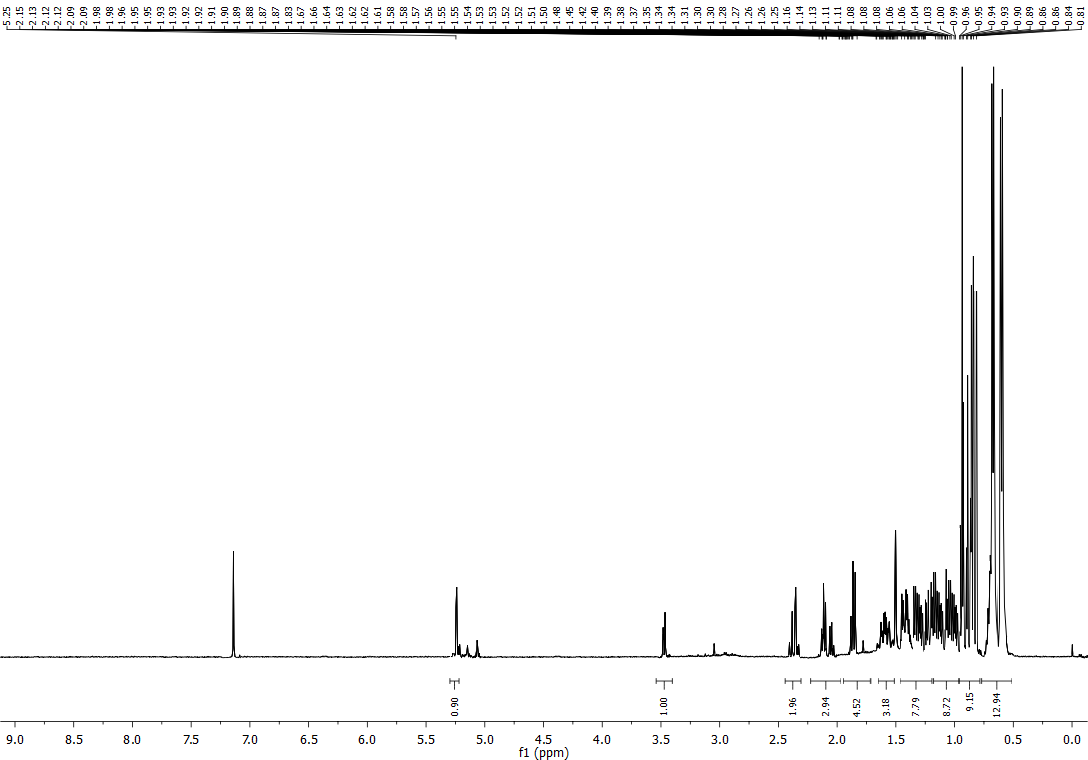


**Figure S1**: ^1^H NMR of Ph-1

**
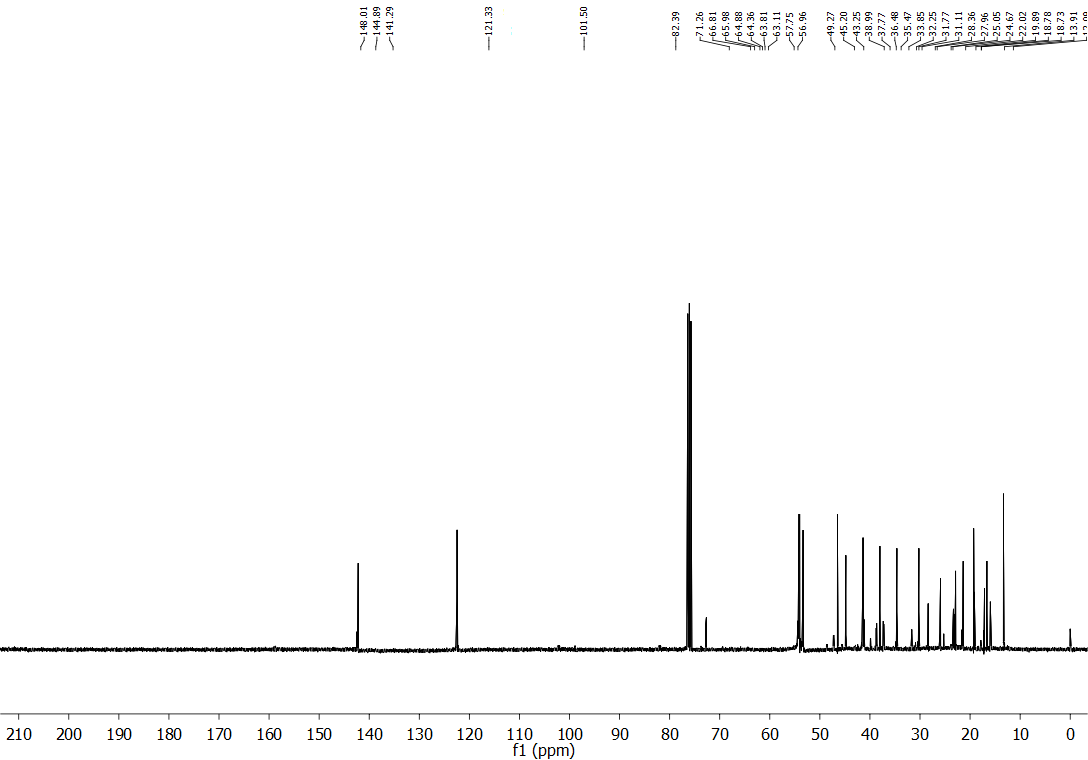
**

**Figure S2**: ^13^C NMR of Ph-1

**
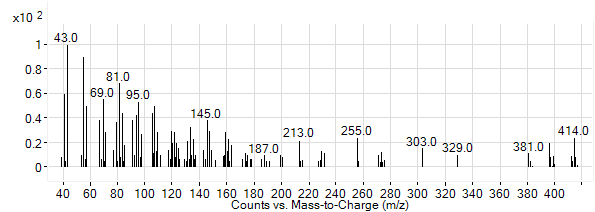
**

**Figure S3:**MS of Ph-1

**Compound Ph-2**

**(E)-17-(5-ethyl-6-methylhept-3-en-2-yl)-10,13-dimethyl-2,3,4,7,8,9,10,11,12,13,14,15,16,17-tetradecahydro-1H-cyclopenta[a]phenanthren-3-ol (Ph-2)**

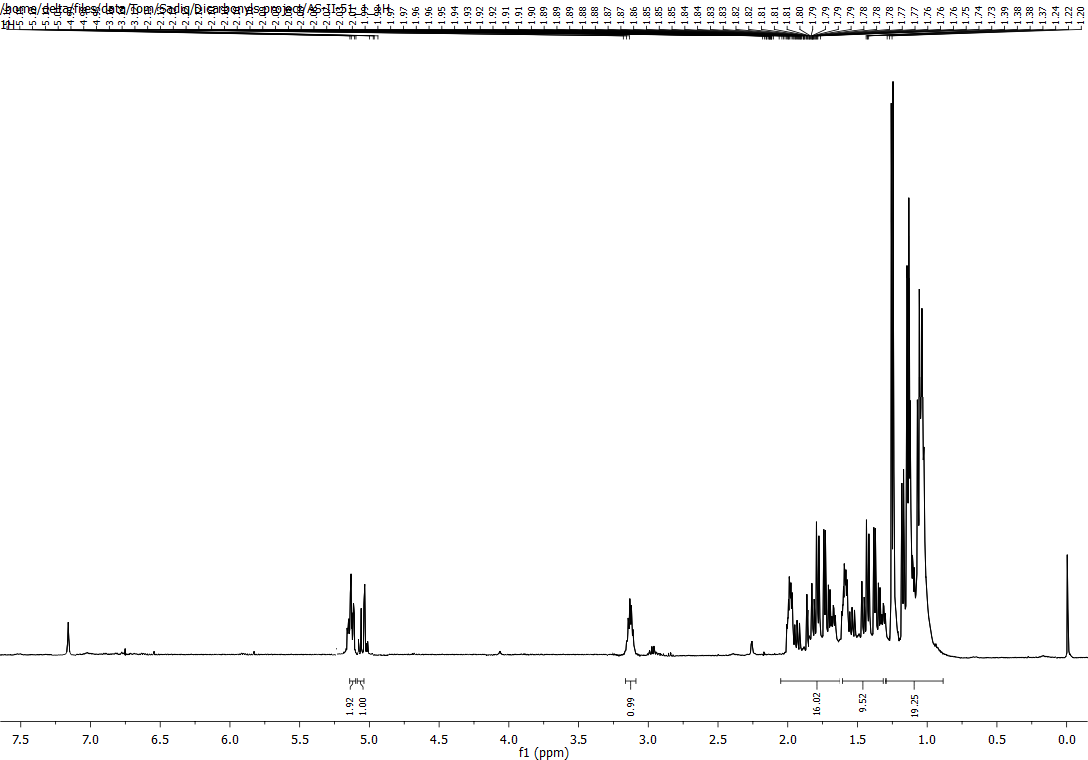


**Figure S4:**^1^H NMR of Ph-2


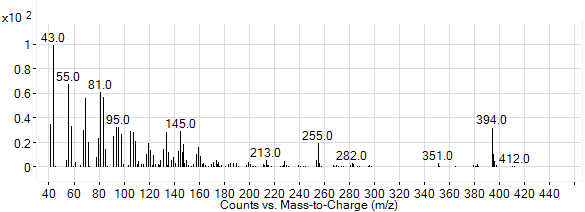


**Figure S5:** MS of Ph-2

**Compound Ph-3**

**4-methyl-5-oxo-tetrahydrofuran-3-yl acetate (Ph-3)**

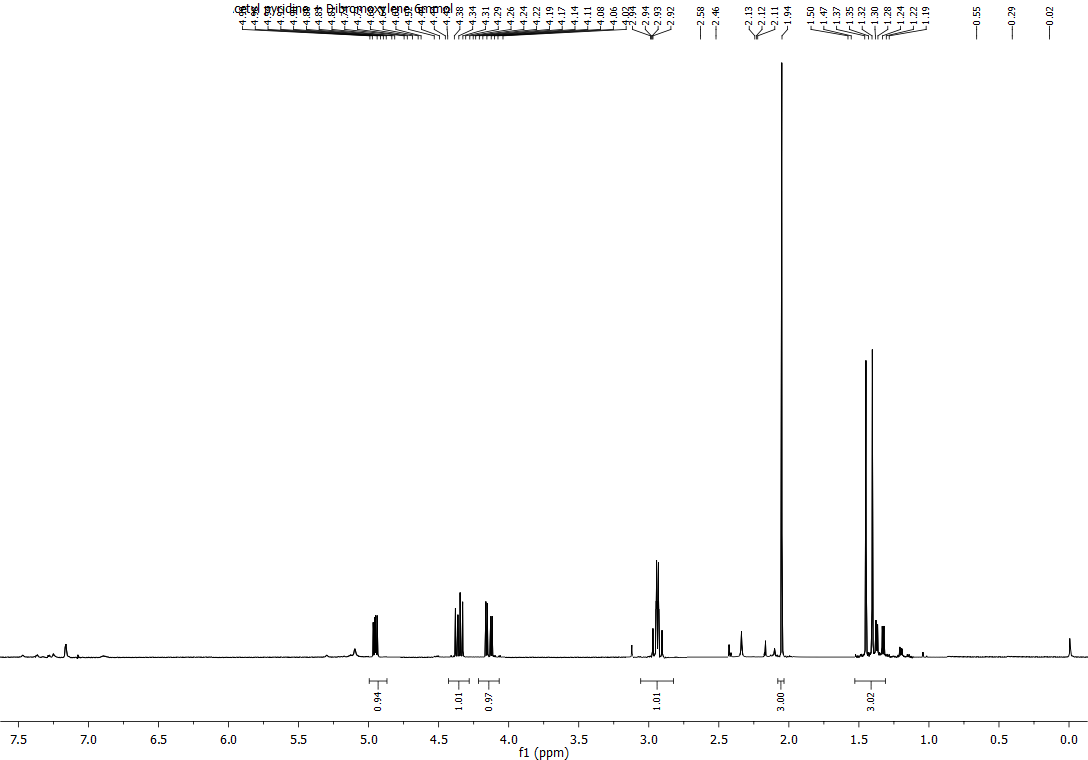


**Figure S6:**^1^H NMR of Ph-3

**
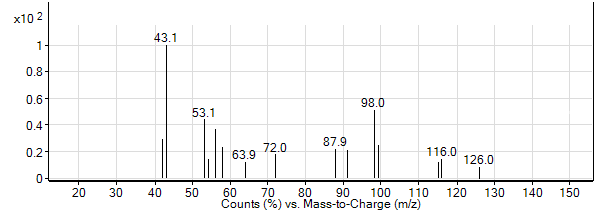
**

**Figure S7:** MS of Ph-3

**Compound Ph-4**

**methyl 4-hydroxy-3-methoxybenzoate (Ph-4)**

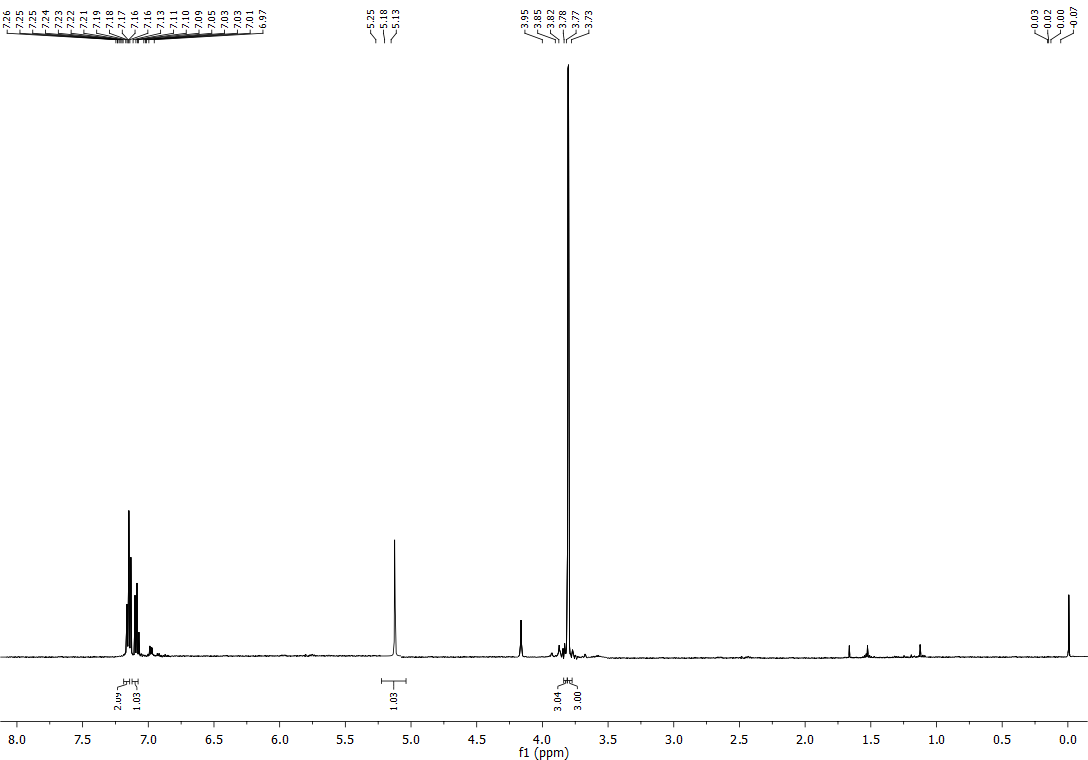


**Figure S8:**^1^H NMR of Ph-4


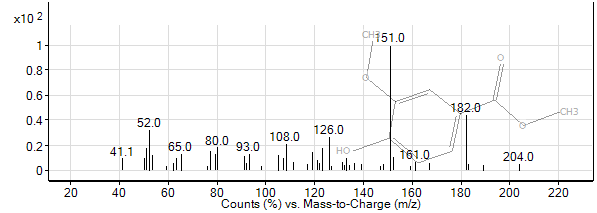


**Figure S9:** MS of Ph-4

**Compound Ph-5**

**1-(3-ethoxy-7-methoxynaphthalen-1-yl)ethanone (Ph-5)**

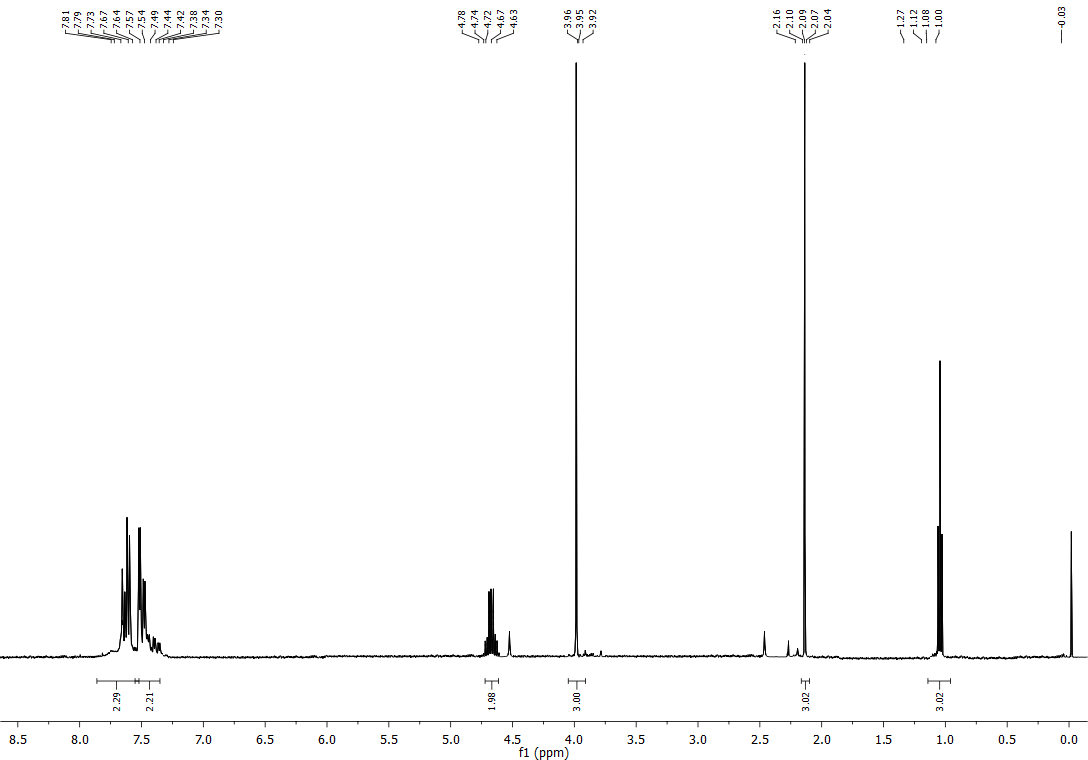


**Figure S10:**^1^H NMR of Ph-5


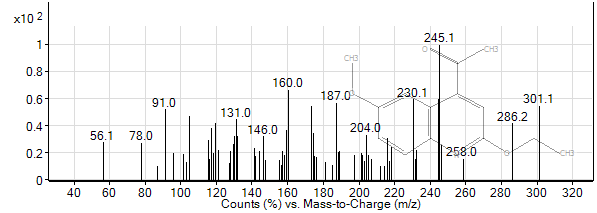


**Figure S11:**MS of Ph-5

**Compound Ph-6**

**(E)-4-methoxy-6-styryl-2H-pyran-2-one (Ph-6)**

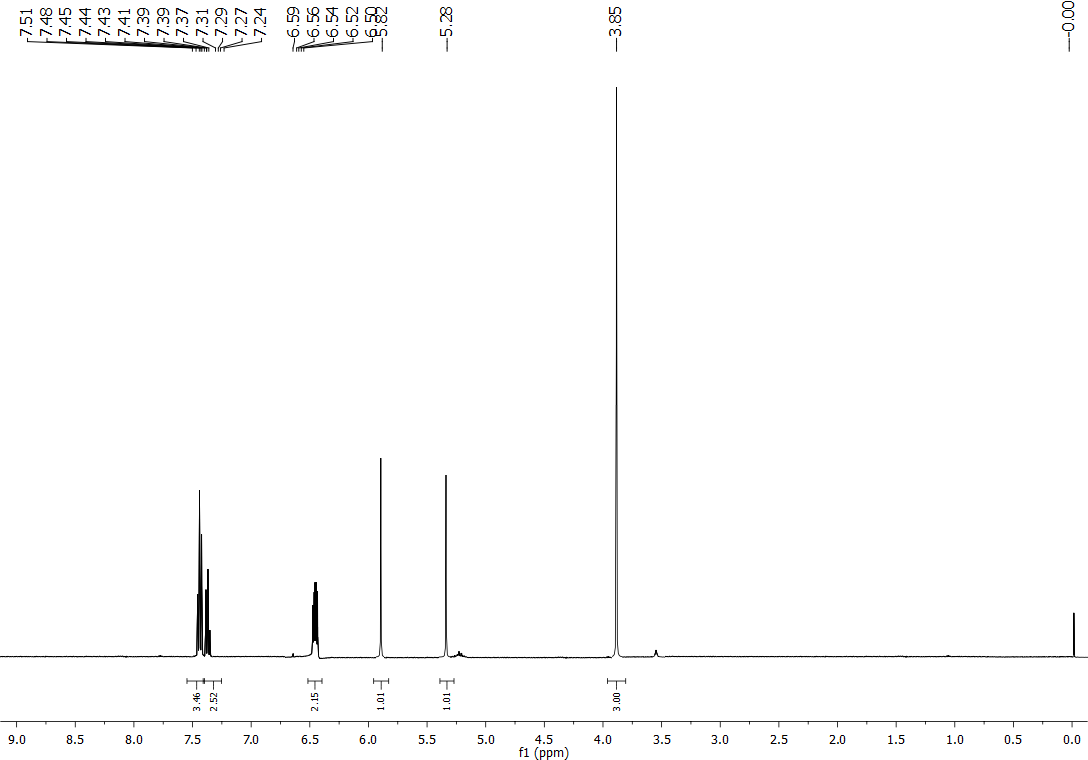


**Figure S12:**^1^H NMR of Ph-6


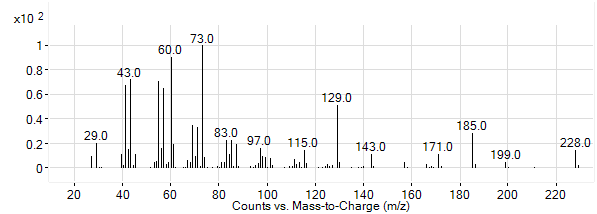


**Figure S13:** MS of PhA-6
